# Novel β-phenylacrylic acid derivatives exert anti-cancer activity by inducing Src-mediated apoptosis in wild-type KRAS colon cancer

**DOI:** 10.1038/s41419-018-0942-x

**Published:** 2018-08-29

**Authors:** Su Jin Kim, Tae Hwan Noh, Sujin Son, Do Hyun Kim, Wooseong Kim, Yunna Lee, Jieun Choo, Gwangbeom Heo, Min Jae Kim, Hae Young Chung, Yunjin Jung, Jee Hyung Jung, Hyung Ryong Moon, Eunok Im

**Affiliations:** College of Pharmacy, Pusan National University, Busan, 46241 Republic of Korea

## Abstract

Many stress conditions including chemotherapy treatment is known to activate Src and under certain condition Src can induce the apoptotic signal via c-Jun N-terminal kinase (JNK) activation. Here we report that the newly synthesized β-phenylacrylic acid derivatives, MHY791 and MHY1036 (MHYs), bind to epidermal growth factor receptor (EGFR) tyrosine kinase domains and function as EGFR inhibitors, having anti-cancer activities selectively in wild-type KRAS colon cancer. Mechanistically, MHYs-induced Src/JNK activation which enhanced their pro-apoptotic effects and therefore inhibition of Src by the chemical inhibitor PP2 or Src siRNA abolished the response. In addition, MHYs generated reactive oxygen species and increased ER stress, and pretreatment with antioxidant-inhibited MHY-induced ER stress, Src activation, and apoptosis. Furthermore, the irreversible EGFR inhibitor PD168393 also activated Src while the reversible EGFR inhibitor gefitinib showed the opposite effect, indicating that MHYs are the irreversible EGFR inhibitor. Collectively, Src can play a key role in apoptosis induced by the novel EGFR inhibitor MHYs, suggesting that activation of Src might prove effective in treating EGFR/wild-type KRAS colon cancer.

## Introduction

Colon cancer represents the third most common malignancy and the fourth leading cause of cancer-related death in the world with the anticipation of increasing incidence rate^1,2^. Despite recent advances in the treatment of colon cancer, chemotherapy is often ineffective and therefore the demand of other approaches is increasing.

Epidermal growth factor receptor (EGFR) is a member of the ErbB family of receptor tyrosine kinases containing an extracellular ligand-binding domain, a transmembrane domain, and an intracellular tyrosine kinase domain^3,4^. Anti-EGFR antibodies are added to the first-line treatment of metastatic colon cancer and this approach elicits more potent anti-tumor effect than conventional chemotherapies^5^. Although the development of an alternative approach is in need for the patients with RAS mutations, more than 55–65% of colon cancer patients who express wild-type KRAS will still benefit from anti-EGFR therapies^6^.

Aberrant activation of the proto oncogene Src enhances cancer progression including colon cancer. Src expression is increased in primary colon adenocarcinoma tissues compared with normal colonic epithelium, and the expression level and activity of Src are further increased in metastatic lesions compared with corresponding primary tumors^7–9^. Therefore, increased expression and activity of Src are the indicative of poor clinical outcomes in colon cancer patients^10^. Notably, the contrasting role of Src has been reported. Constitutively active v-Src can induce apoptosis in rat fibroblasts when Ras and PI3K are inhibited^11^. Additionally, c-Src regulates estrogen-induced apoptosis in breast cancer cells^12^. As the mechanism by which Src induces apoptosis, Src-mediated JNK activation was suggested. The JNK activation can regulate the intrinsic mitochondrial apoptosis and is essential for apoptosis induced by anti-cancer agents^13–16^.

Previous studies showed that thioxoimidazolidinone derivatives exerted chemopreventive potential on hamster buccal pouch carcinogenesis and a potent anti-cancer activity to prostate cancer cells^17,18^. Furthermore, ethyl pyruvate and its functional analog 2-acetamidoacrylate inhibited tumor growth in mice^19^. Within this context, the newly synthesized β-phenylacrylic acid derivatives can be considered anti-cancer agents, since they contain 2-thioxoimidazolidin-4-one or 2-acetamido acrylic acid moiety. Here we report the development of novel EGFR inhibitors, having pro-apoptotic and tumor suppressive effects in wild-type KRAS colon cancer. These agents induce apoptosis by generating reactive oxygen species (ROS) and activating the Src-JNK signaling axis.

## Results

### The β-phenylacrylic acid derivatives, MHY791 and MHY1036 (MHYs), induce mitochondria-mediated apoptosis

**Fig. 1:**
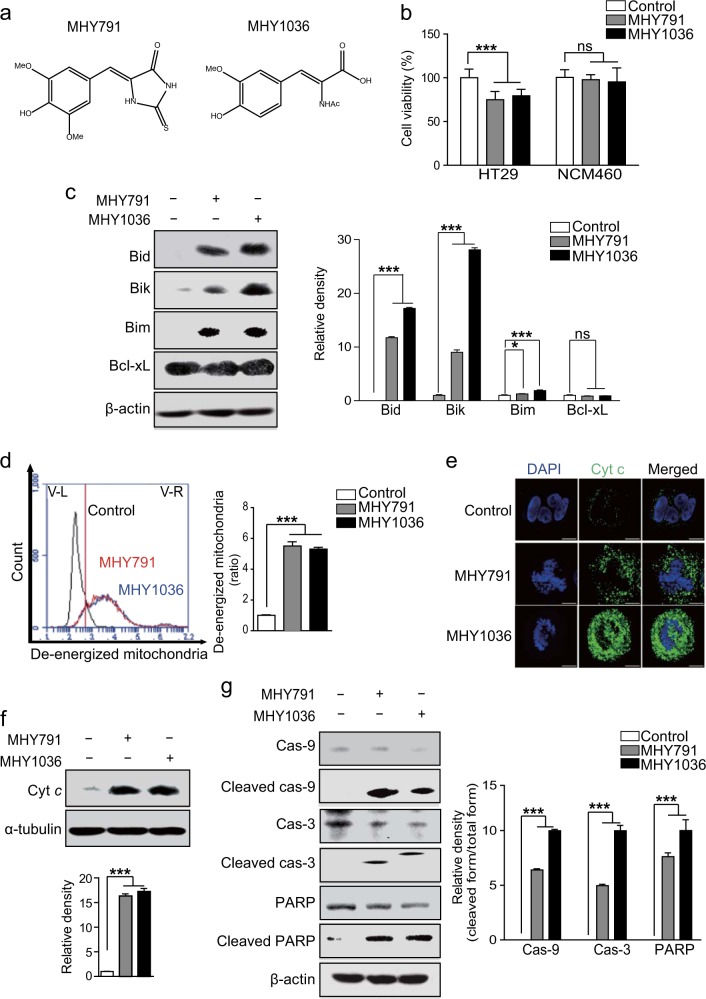
MHYs induce mitochondrial apoptosis in human colon cancer cells. **a** The structures of MHY791 and MHY1036 are indicated. **b** HT29 and NCM460 cells were treated with MHYs (10 μM) for 24 h and their viabilities were measured by MTT assays (*n* = 5). Data presented as a percent of control cells. **c** HT29 cells were treated with MHYs (10 μM) for 24 h and the protein levels of apoptotic markers were evaluated by immunoblot analyses. **d** HT29 cells were treated with MHYs (10 μM) for 24 h and FACS analysis was performed to investigate mitochondrial membrane dysfunction (*n* = 3). **e** Release of Cyt *c* into cytoplasm as indicated by green fluorescence staining was observed in HT29 cells treated with MHYs (10 μM, 24 h). Scale bars, 300 μm. **f** Cyt *c* levels in total cell lysate of HT29 cells treated with MHYs (10 μM, 24 h) were measured. **g** Cleaved caspase-9, caspase-3, and PARP levels were evaluated in HT29 cells treated with MHYs (10 μM, 24 h). Relative density measurements correspond to the intensities of the immunoblotting bands normalized to an internal control (*n* = 3). Data are shown as mean ± SD. Statistical significance was determined by one-way analysis of variance (ANOVA) followed by Bonferroni’s post-hoc test. Statistical significance is indicated as *p < 0.05, ***p < 0.0001. The ns indicates that the comparison was not statistically significant

Chemical compounds with 2-thioxoimidazolidinone or 2-acetamidoacrylate moiety showed anti-cancer effects in previous studies^17–20^. Newly synthesized β-phenylacrylic acid derivatives, MHYs, also contain 2-thioxoimidazolidinone or 2-acetamidoacrylate moiety, respectively, suggesting these compounds may exert anti-cancer activity (Fig. 1a). To test this hypothesis, we investigated cytotoxic effects of MHYs on colon cancer cells. MHYs reduced the cell viability of the human colon adenocarcinoma cell line HT29, while they did not affect the viability of the normal colonic epithelial cell line NCM460 (Fig. 1b). Numerous cytotoxic stimuli including chemotherapeutic agents are known to induce the intrinsic pathway of apoptosis in which the mitochondrial pathway is involved^21,22^. Consistent with this finding, the expression levels of the pro-apoptotic Bid, Bik, and Bim were increased by the treatment with MHYs, while the level of the anti-apoptotic Bcl-xL remained unchanged (Fig. 1c). As increased pro-apoptotic/anti-apoptotic Bcl-2 family ratio can lead to mitochondrial membrane dysfunction and increase mitochondrial membrane permeabilization^23^, we measured mitochondrial membrane potential in HT29 cells treated with MHYs. As shown in Fig. 1d, the population of the cells with de-energized mitochondria shown by fluorescence-activated cell sorting (FACS) analysis was increased upon MHY treatment. Given that the disruption of mitochondrial outer membrane triggers the release of Cyt *c* into the cytosol^24^, Cyt *c* release was analyzed by immunofluorescence staining and immunoblotting. Cyt *c* release was increased by MHYs as indicated by increased intensity of the green fluorescence signal and this was accompanied by formation of apoptotic bodies in the nucleus (Fig. 1e). In the cytosolic fraction of cell lysates, Cyt *c* release was dramatically increased by MHYs compared to controls (Fig. 1f). Consecutively, released Cyt *c* forms the apoptosome complex of Cyt *c*/Apaf-1/caspase-9 and triggers the activation of caspase-3, which then cleaves substrates and executes various apoptotic events^25^. In agreement with this notion, the levels of cleaved caspase-9, caspase-3, and PARP were increased by MHYs compared to control, while the levels of full-length caspase-9, caspase-3, and PARP were decreased by MHYs (Fig. 1g). Taken together, these data suggest that MHYs confer anti-cancer activity by inducing cell death via the mitochondrial pathways of apoptosis.

### MHYs suppress the tumor growth in the mouse xenograft model of colon cancer

**Fig. 2:**
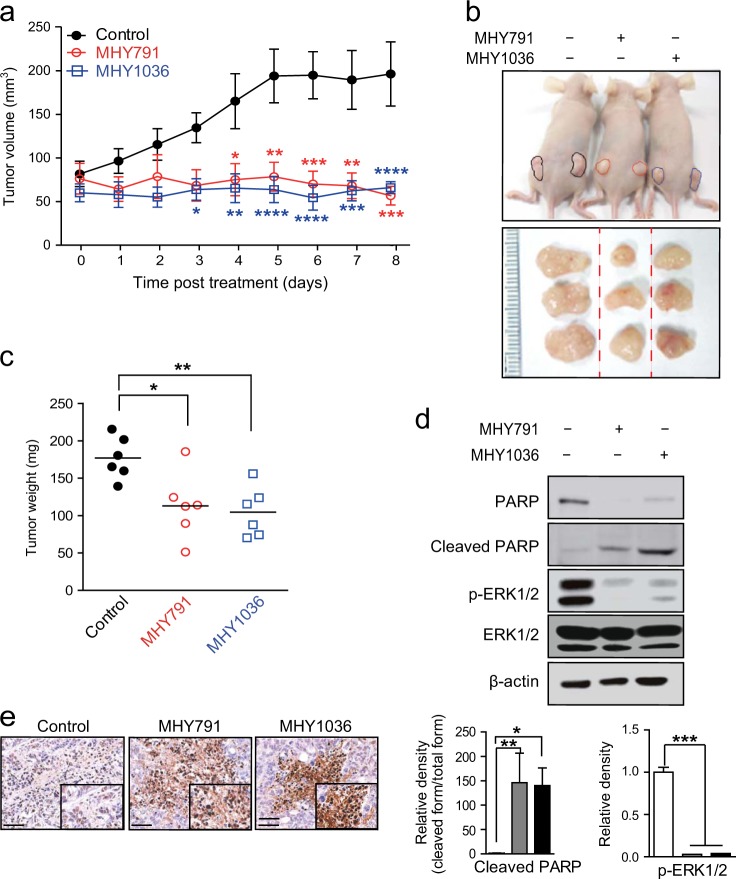
MHYs inhibit tumor progression in a mouse xenograft model. **a** At 7 days after HT29 cell inoculation, mice harboring human colon tumors were treated with vehicle (control) or MHYs (3 mg/kg) for additional 8 days. Tumor growth was measured in control-treated and MHY-treated groups (*n* = 8 control, *n* = 5 MHY791, *n* = 6 MHY1036). **b** Representative tumor image of each treatment group. **c** Tumor weight was measured (*n* = 6). **d** Cleaved PARP and phosphorylation of ERK1/2 levels were evaluated in MHY-treated xenografts. Relative density measurements correspond to the intensities of the immunoblotting bands normalized to an internal control (*n* = 3). **e** TUNEL assays were performed in MHY-treated xenografts. Scale bar, 50 μm. Data are shown as mean ± SEM. Statistical significance was determined by two-way ANOVA followed by Bonferroni’s post-hoc test. Statistical significance is indicated as *p < 0.05, **p < 0.01, ***p < 0.001 ****p < 0.0001

In order to address if MHYs confer anti-cancer activities in an in vivo model of colon cancer, HT29 cells were subcutaneously inoculated into the flanks of female nude mice. When the size of tumors reached 50 mm^3^, the daily peritumoral injection of MHYs started. MHY-treated tumors grew smaller and slower than vehicle-treated tumors, resulting in substantially reduced tumor volume in MHY-treated tumors (Fig. 2a). Additionally, tumor size and weight were markedly decreased in MHY-treated tumors than control tumors (Fig. 2b, c). In MHY-treated tumors, cleaved PARP level was increased but full-length PARP level was decreased. These data suggest that MHYs-induced apoptosis (Fig. 2d). Phosphorylation of ERK1/2 was decreased by MHYs in xenografts (Fig. 2d). Furthermore, the number of apoptotic cells detected by TUNEL staining was increased in MHY-treated tumors than vehicle-treated tumors (Fig. 2e). Taken together, these data suggest that MHYs suppress the tumor growth in mice by inducing apoptosis.

### MHYs inhibit EGFR activation and its downstream signaling pathways

**Fig. 3:**
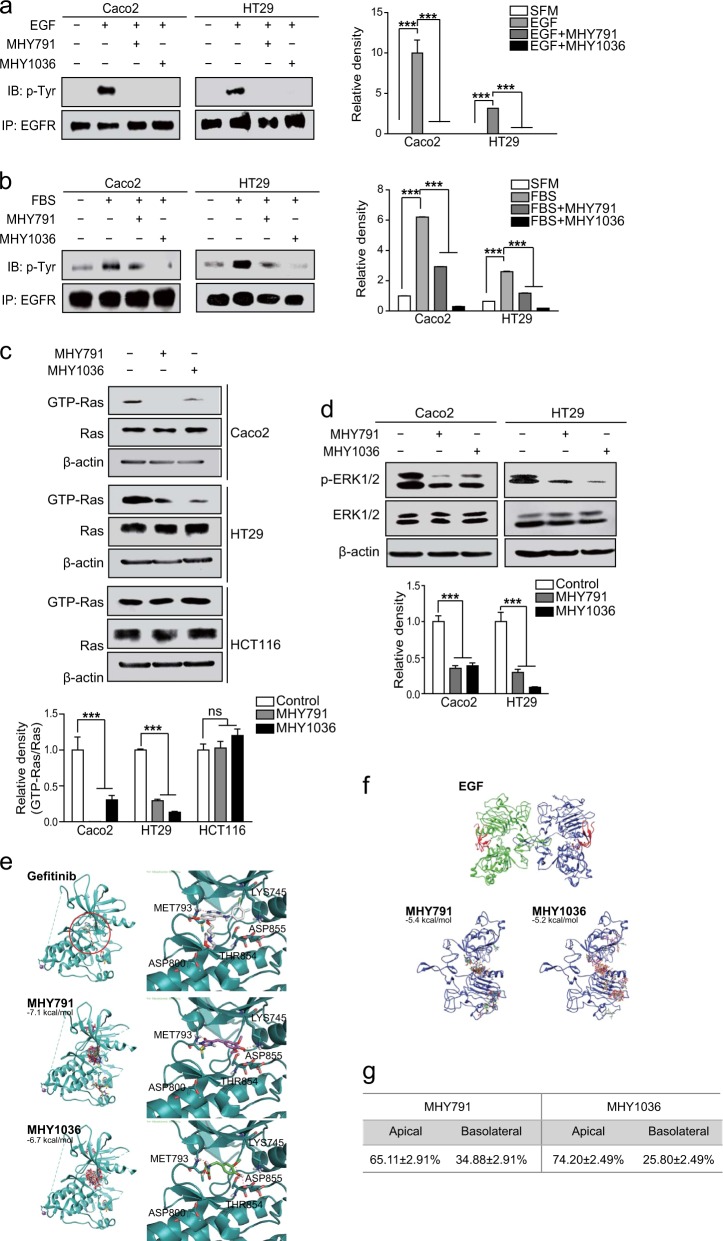
Interaction between MHYs and EGFR inhibits the EGFR activity. **a**, **b** Caco2 and HT29 cells were pre-treated with MHYs (10 μM) for 30 min followed by EGF (100 ng/ml) or 10% (v/v) FBS for 5 min. Total cell lysates were immunoprecipitated with EGFR antibodies and then immnoblotted with phosphor-Tyr antibodies. SFM serum-free medium; Tyr tyrosine. **c** Caco2, HT29, and HCT116 cells were treated with MHYs (10 μM) for 24 h and protein lysates were subjected to Ras activation assays. The ns indicates that the comparison was not statistically significant. **d** The level of ERK1/2 phosphorylation was measured in Caco2 and HT29 cells treated with MHYs (10 μM, 24 h). Relative density measurements correspond to the intensities of the immunoblotting bands normalized to an internal control (*n* = 3). Data are shown as mean ± SD. ***p < 0.001. **e** Docking simulation of gefitinib to the Tyr kinase domain of EGFR (PDB code number 4WRG) (top). Simulation of MHY791 to the Tyr kinase domain of EGFR (PDB code number 4WKQ) (middle). Simulation of MHY1036 to the Tyr kinase domain of EGFR (PDB code number 4WKQ) (bottom). **f** Docking simulation of EGF (top), MHY791 (bottom left), and MHY1036 (bottom right) to the extracellular domain of EGFR (PDB code number 1MOX). **g** MHYs (100 μM) were added to the apical side of Caco2 cell monolayer and incubated for 24 h. Concentrations of MHYs in the apical and the basolateral sides were measured (*n* = 3). Data are shown as mean ± SEM

Our data suggest that MHYs promote cell death in HT29 cells and thereby contributes to the tumor regression in mice. We next tested whether MHYs could inhibit the tumor growth in a range of human colon cancer cell lines. To address this, we investigated if MHYs regulate the cell viability of a series of colon cancer cell lines including Caco2, DLD1, and HCT116. Similar to the reduced cell viability in HT29 cells, MHYs reduced the viability of Caco2 cells. In contrast, the viabilities of DLD1 and HCT116 cells were not changed in response to MHYs (Supplementary Fig. 1a, b). To investigate this further, colon cancer cell lines are then distinguished by the expression status of KRAS whose oncogenic mutations are found in 30–50% of colon cancer patients^26^. It is noteworthy that Caco2 and HT29 cells express wild type KRAS, whereas HCT116 and DLD1 cells express mutated KRAS (G → D)^27^. Having found that MHYs inhibited the tumor growth only in Caco2 and HT29 cells, it is possible that the KRAS status is correlated with resistance to MHYs and in other words, MHYs require wild type KRAS to exert their anti-cancer effects. Moreover, KRAS mutation predicts lack of response to EGFR inhibitors^28–30^. Therefore, we hypothesized that MHYs could act as EGFR inhibitors. To prove this hypothesis, we first tested if MHYs inhibit the EGFR activity in Caco2 and HT29 cells which express EGFR^31^. Activation of EGFR by EGF (100 ng/ml) or 10% FBS treatment was inhibited by MHYs in Caco2 and HT29 cells (Fig. 3a, b).

Since Ras and ERK1/2 are the signaling molecules participating EGFR-mediated signaling pathways, we next examined whether the activation of Ras and ERK1/2 would be inhibited by MHYs. Concomitant to reduced EGFR activity, we observed that the activities of Ras and ERK1/2 were also inhibited by MHYs. Ras activation represented by increased GTP-bound form of Ras was inhibited by MHYs in Caco2 and HT29 cells but not in KRAS mutant HCT116 cells (Fig. 3c). Similarly, phosphorylation of ERK1/2 was inhibited by MHYs in Caco2 and HT29 cells (Fig. 3d). Together, these data suggest that MHYs inhibit EGFR-mediated signaling pathways in colon cancer cells expressing wild type KRAS.

### MHYs bind to the intracellular domain of EGFR and inhibit EGFR activation

To investigate the mechanism by which MHYs inhibit the EGFR activity, we examined the probability that MHYs directly interact with EGFR to inhibit its activity by conducting the computerized docking simulation. On the preferential basis, EGFR domains were classified into extracellular ligand-binding domain and intracellular Tyr kinase domain. EGF binds to extracellular domain of EGFR, whereas gefitinib, a well-known EGFR inhibitor^32^, binds to Tyr kinase domain. Docking simulation of MHYs to EGFR Tyr kinase domain exhibited that they bind to the same site with the gefitinib binding. This gefitinib-binding site is well known as the general binding site of many EGFR Tyr kinase inhibitors. MHYs also showed similar binding topology as that of gefitinib in the binding domain. The relative docking affinities of MHY791 (−7.1 kcal/mol) and MHY1036 (−6.7 kcal/mol) to Tyr kinase domain were close to that of gefitinib (−8.5 kcal/mol) (Fig. 3e). In the case of gefitinib, the nitrogen of quinazoline moiety makes a hydrogen bond with the backbone amide proton of Met793 of Tyr kinase domain and the oxygen of morpholine moiety forms a hydrogen bond with the carboxylic proton of Asp800 residue (Fig. 3e top). Similarly, MHY791 makes hydrogen bonds with backbone of Met793. The carbonyl group of thiohydantoin moiety interacts with the backbone amide proton of Met793, and the NH of thiohydantoin moiety forms a hydrogen bond with the backbone carbonyl group of Met793 (Fig. 3e middle). The carboxyl group of MHY1036 also makes a hydrogen bond with the backbone amide proton of Met793 (Fig. 3e bottom). However, docking simulation of MHYs with the extracellular domain displayed irregular patterns of binding and their affinities (around −5.4 to −5.2 kcal/mol) were weak (Fig. 3f). It is speculated that MHYs have low chances to bind to the extracellular domain of EGFR. Collectively, our data suggest that the binding affinity and topology of MHYs to the Tyr kinase domain of EGFR are comparable to those of gefitinib.

These findings prompted us to test whether MHYs were cell permeable and thus could bind to an intracellular target. To test this notion, the monolayer culture of Caco2 cells was treated with MHYs (100 μM) in the apical side for 24 h, followed by evaluating MHY concentrations in the apical and the basolateral sides. Notably, 34.88% (MHY791) and 25.80% (MHY1036) of the apically treated MHYs were identified in the basolateral side of the monolayer culture (Fig. 3g), suggesting that MHYs are likely to cross cell membrane. Taken together, these data suggest that MHYs have high chances to bind to the intracellular tyrosine kinase domain of EGFR, resulting in the inhibition of its intracellular signaling pathways.

### Phosphorylation of Src is a key event in the JNK-mediated apoptosis by MHYs

**Fig. 4:**
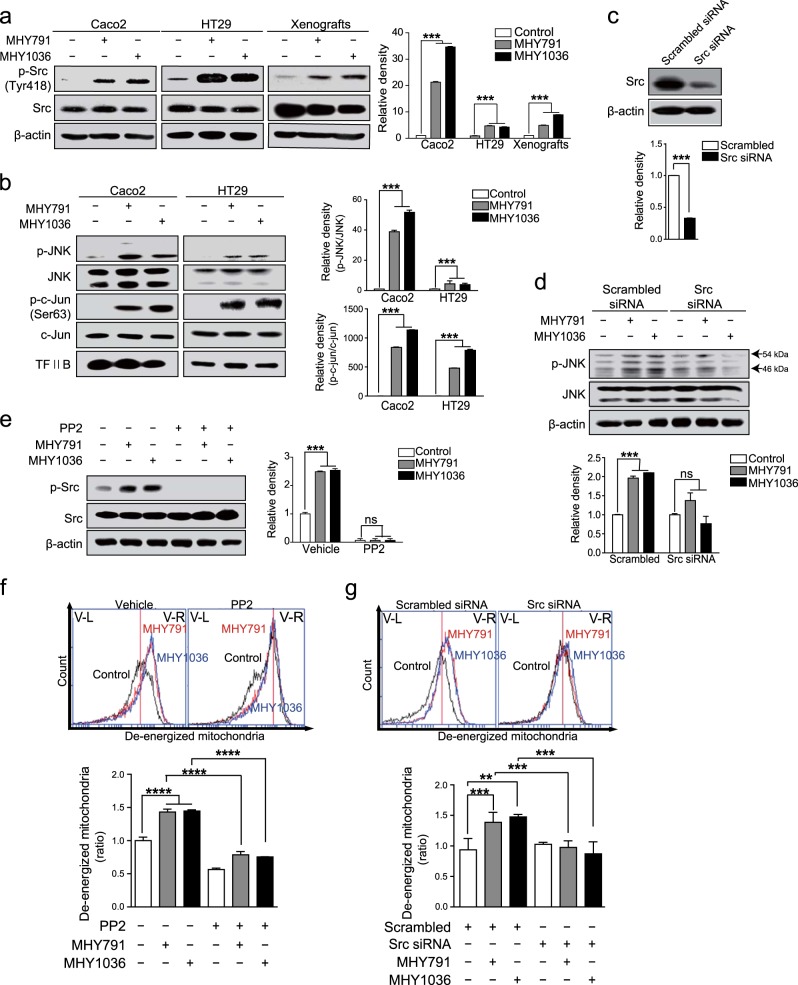
Activation of Src mediates MHY-induced apoptosis. **a** Caco2 and HT29 cells were treated with MHYs (10 μM) for 24 h and xenografts (HT29 cells) were treated with MHYs (3 mg/kg) for 8 days. Src phosphorylation levels were measured in Caco2 and HT29 cells as well as in xenografts. **b** Caco2 and HT29 cells were treated with MHYs (10 μM) for 24 h to investigate the levels of phospho-JNK and phospho-c-Jun. **c** HT29 cells were transfected with scrambled siRNA and Src siRNA. **d** HT29 cells expressing scrambled siRNA and Src siRNA were treated with MHYs (10 μM) for 24 h to measure JNK phosphorylation. **e** HT29 cells were pre-treated with the Src inhibitor PP2 (100 nM) for 30 min followed by MHYs (10 μM) for 24 h. Relative density measurements correspond to the intensities of the immunoblotting bands normalized to an internal control (*n* = 3). **f** Mitochondrial membrane dysfunction was determined by FACS analysis. HT29 cells were pre-treated with PP2 (100 nM) for 30 min followed by MHYs (10 μM) for 24 h (*n* = 3). **g** HT29 cells expressing scrambled siRNA and Src siRNA were treated with MHYs (10 μM) for 24 h to measure mitochondrial dysfunction (*n* = 3). Data are shown as mean ± SD. Statistical significance was determined by one-way ANOVA followed by Tukey post-hoc test. Statistical significance is indicated as **p < 0.01, ***p < 0.001, ****p < 0.0001

The activation of Src is able to induce apoptosis in colon cancer cells^33^. In addition, Src activation increases the JNK activity, which subsequently induces the mitochondrial apoptotic pathways^13,14^. Within this context, we next questioned whether MHYs have a potential to induce Src activation in colon cancer cells, thereby make a contribution to induction of apoptosis. Surprisingly, Src activation evaluated by the phosphorylation of Src at Tyr418 was increased by MHYs in Caco2 and HT29 cells as well as xenograft tumors (Fig. 4a). Given the capability of activated Src of inducing JNK activation, we next examined whether MHYs regulate the JNK activity. Phosphorylations of JNK and c-Jun at Ser63 were increased by MHYs (Fig. 4b), suggesting the intriguing possibility that the Src/JNK pathway might be involved in MHY-mediated apoptosis. To test whether MHY-induced JNK activation is regulated by Src, we used Src siRNA approach. The expression level of Src was reduced by siRNA and this reduction blocked MHY-induced JNK phosphorylation in HT29 cells (Fig. 4c, d). To investigate this further, we examined MHY-induced mitochondrial dysfunction after pretreatment with the Src tyrosine kinase inhibitor PP2 or transfection of Src siRNA. In addition to inhibiting MHY-mediated Src phosphorylation, PP2 notably reduced de-energized mitochondrial fraction indicating an anti-apoptotic role of PP2 in MHY-treated colon cancer cells (Fig. 4e, f). Similarly, Src siRNA diminished MHY-mediated mitochondrial dysfunction (Fig. 4g). Taken together, our data imply that MHYs might utilize the Src-dependent signaling pathway to exhibit their tumor suppressive effect through the induction of apoptosis in colon cancer cells.

### Similar to MHYs, the irreversible EGFR inhibitor PD168393 activates Src in wild type KRAS colon cancer cells

**Fig. 5:**
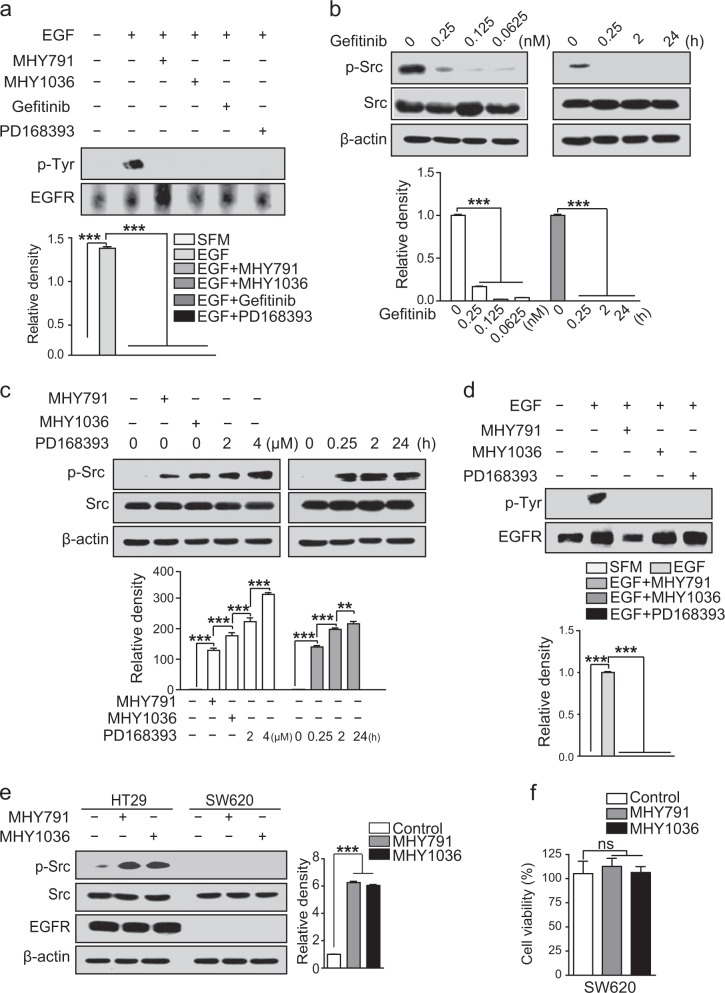
Irreversible inhibition of EGFR induces Src activation. **a** HT29 cells were pre-treated with MHYs (10 μM), gefitinib (0.25 nM), or PD168393 (2 μM) for 1 h followed by EGF (100 ng/ml) for 5 min. Total cell lysates were immunoprecipitated with EGFR antibodies and then immnoblotted with phospho-Tyr antibodies. **b** HT29 cells were pre-treated with gefitinib at indicated concentrations and time points to measure Src phosphorylation. **c** HT29 cells were pre-treated with PD168393 at indicated concentrations and time points. Cells were treated with MHYs (10 μM) for 24 h. **d** HT29 cells were pre-treated with MHYs (10 μM) or PD168393 (2 μM) for 1 h and then were washed twice in warm SFM to remove MHYs or PD168393. This process was repeated every 2 h until 6 h from the first wash. Cells were then stimulated with EGF (100 ng/ml) for 5 min. SFM serum-free medium. **e** HT29 and SW620 cells were treated with MHYs (10 μM) for 24 h to measure Src phosphorylation. Relative density measurements correspond to the intensities of the immunoblotting bands normalized to an internal control (*n* = 3). Data are shown as mean ± SD. **p < 0.01, ***p < 0.001. **f** SW620 cells were treated with MHYs (10 μM) for 24 h and their viabilities were measured by MTT assays (*n* = 3). Data presented as a percent of control cells. Statistical significance was determined by one-way ANOVA followed by Bonferroni’s post-hoc test. The ns indicates that the comparison was not statistically significant

Our data suggested that MHYs bind to the cytoplasmic kinase domain of EGFR and inhibit its receptor kinase activity (Fig. 3). In parallel, MHYs induce Src activation to mediate subsequent JNK activation (Fig. 4). Given that the pro-apoptotic effect of MHYs on colon cancer cells is derived by the Src/JNK signaling axis, it is reasonable to speculate that inhibition of EGFR and activation of the Src/JNK signaling pathway appear to cooperatively participate in enhancing the cell death. However, previous studies suggested that Src activation can increase cancer cell proliferation and metastasis of colon cancer. Accordingly, active Src is considered a biomarker of CRC metastasis and poor prognosis and therefore combined EGFR and Src inhibition has been evaluated as optimal chemotherapy in colon cancer^8,10,34–36^. To reconcile such discrepancy, we investigated whether other EGFR inhibitors, such as gefitinib and PD168393 can activate Src. As expected, pretreatment with gefitinib or PD168393 for 1 h prior to EGF treatment inhibited EGF-induced phosphorylation of EGFR in HT29 cells (Fig. 5a). Moreover, the reversible EGFR inhibitor gefitinib inhibited endogenous Src activation in a dose-dependent and time-dependent manner (Fig. 5b), while the irreversible EGFR inhibitor PD168393 increased Src activation and the increased level of Src activation by PD168393 was comparable to that by MHYs (Fig. 5c). Based on these findings, we hypothesized that irreversible inhibition of EGFR is required for Src activation and thus MHYs act as irreversible EGFR inhibitors. To understand the kinetics of MHYs, we used a wash-out approach. The inhibitory effects of MHYs and PD168393 on the EGFR activity was still evident at 6 h after wash-out, indicating that similar to PD168393, MHYs are irreversible EGFR inhibitors (Fig. 5d).

We next questioned whether EGFR expression is prerequisite to the effect of MHYs on Src activity. To address this, we tested whether MHYs can induce Src activation in EGFR null colon cancer cells (SW620). While MHYs elevated Src activation in EGFR-positive HT29 cells, they failed to activate Src in SW620 cells (Fig. 5e). Notably, the expression levels of total Src are preserved in HT29 and SW620 cells. In line with no activation of Src, we did not find a significant change in the viability of SW620 cells by MHYs (Fig. 5f). Taken together, our data suggest that MHYs act as irreversible EGFR inhibitors and their anti-cancer effects including activation of Src and induction of apoptosis may require prior inhibition of EGFR.

### ROS production and PERK phosphorylation are increased by MHYs

**Fig. 6:**
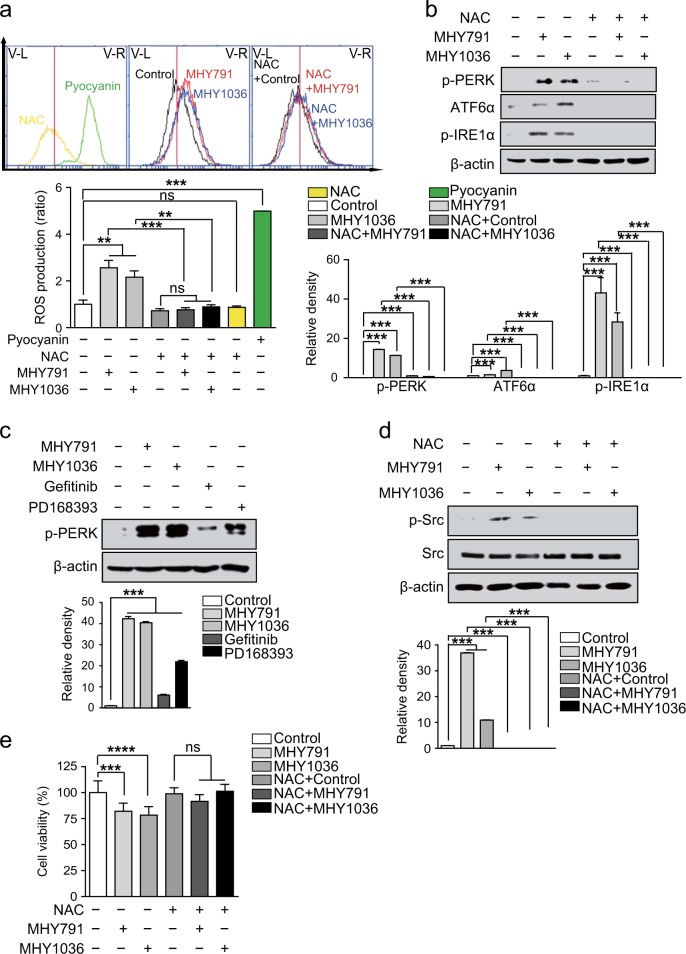
ROS production and ER stress are increased by MHYs. **a** HT29 cells were treated with NAC (5 mM), MHYs (10 μM), or pyocyanin (200 µM, a positive control) for 1 h and ROS production was measured by FACS analysis. Data are presented as ratios relative to the vehicle control. **b** HT29 cells were pre-treated with NAC (5 mM) for 30 min followed by MHYs (10 μM) for 24 h and then the expression levels of ER stress marker proteins were evaluated. **c** HT29 cells were treated with MHYs (10 μM), gefitinib (0.25 nM), or PD168393 (2 μM) for 24 h to measure PERK phosphorylation. **d** Src phosphorylation levels were measured in HT29 cells pre-treated with NAC (5 mM, 30 min) and then treated with MHYs (10 μM, 24 h). Relative density measurements correspond to the intensities of the immunoblotting bands normalized to an internal control (*n* = 3). Data are shown as mean ± SD. **e** HT29 cells were pre-treated with NAC **(**5 mM) for 30 min followed by MHYs (10 μM) for 24 h and then cell viabilities were measured by MTT assays. Data presented as a percent of control cells. Statistical significance was determined by one-way ANOVA followed by Bonferroni’s (**a**, **b**) or Tukey (**f**) post-hoc test. Statistical significance is indicated as **p < 0.01, ***p < 0.001, ****p < 0.0001. The ns indicates that the comparison was not statistically significant

We next examined the effector molecule mediating the cell death responses derived by MHYs. It has been previously reported that ROS or oxidative stress induces Src activation and they can also trigger apoptotic cell death in various settings^36–40^. Moreover, EGFR inhibitors, including monoclonal antibodies and tyrosine kinase inhibitors, were reported to induce oxidative stress in various cancer cells; thereby potentiating anti-tumor efficacy of those inhibitors^41^. Furthermore, when ROS is produced inside cells, Src activates JNK-mediated apoptosis pathway^42^. Therefore, it is conceivable that MHYs would induce ROS production, which might be involved in subsequent Src activation and apoptosis. To test this, we first investigated whether MHYs induce ROS production in HT29 cells. Treatment with MHYs for 1 h significantly increased ROS production when evaluated by FACS, and the amount of ROS produced by MHYs was comparable to that by the positive control pyocyanin (Fig. 6a). Co-treatment of antioxidant N-acetyl-l-cysteine (NAC, 5 mM) inhibited MHY-induced ROS production (Fig. 6a).

In addition, it has been previously reported that activation of Src is linked to endoplasmic reticulum (ER) stress and the JNK signaling pathways mediate ER stress-induced apoptosis^43–45^. We, therefore, investigated further whether MHY-induced Src activation is associated with ER stress. Levels of ER stress markers p-PERK, p-IRE1α, and ATF6α were increased by MHYs but pre-treatment with NAC (5 mM) for 30 min effectively inhibited MHY-induced ER stress as shown by reduction in IRE1α and PERK phosphorylation and ATF6α expression (Fig. 6b). Moreover, PERK phosphorylation was increased by MHYs and PD168393, while it was barely increased by gefitinib (Fig. 6c). To develop these findings further, we next questioned whether inhibition of ER stress by NAC could block MHY-mediated cell death and reverse Src activation. Indeed, pretreatment with NAC effectively inhibited Src activation induced by MHYs (Fig. 6d). Furthermore, MHY-induced apoptosis was reversed by NAC as shown by improved cell viability (Fig. 6e). Taken together, these data clearly demonstrate that MHYs induce ROS production to elicit ER stress, and subsequently this triggers Src activation, resulting in Src-dependent apoptosis in colon cancer cells.

## Discussion

**Fig. 7:**
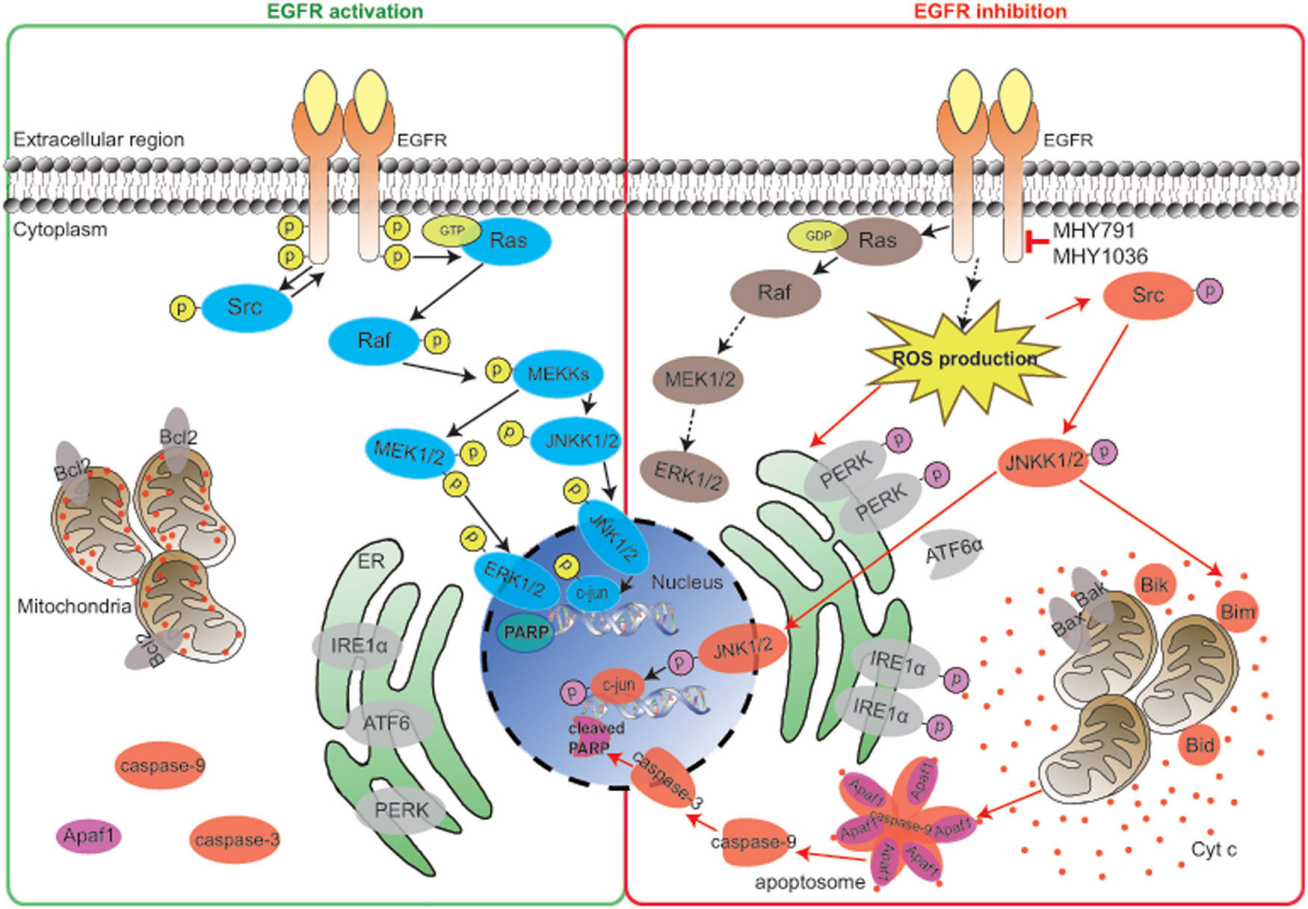
Proposed mechanism of actions of MHYs . Graphical summary shows a putative mechanism of anti-cancer effects of MHYs with the proposed model of Src-mediated apoptosis

Our data reveal that *β*-phenylacrylic acid derivatives, MHYs, function as novel EGFR inhibitors in KRAS wild-type colon cancer. Especially, this anti-cancer effect is mediated by the inactivation of Ras/ERK and the activation of Src. Mechanistically, MHY-mediated ROS production and ER stress trigger Src/JNK-mediated apoptosis via the mitochondrial pathways (Fig. 7).

Results from docking simulation suggest that MHYs bind to the EGFR Tyr kinase domain, which is the general binding site of many EGFR Tyr kinase inhibitors (Fig. 3d). Subsequent studies showed reduction of EGFR phosphorylation and inhibition of RAS and ERK activation by MHYs supporting their role as EGFR inhibitors (Fig. 3a, b, c). However, MHYs appear to be incompatible to the colon cancer with *RAS* mutations because MHYs suppressed the growth of colon cancer cells with only wild-type KRAS (Fig. 1b and Supplementary Fig. 1).

On the other hand, other frequent mutations observed in colon cancer cell lines include *PI3KCA* and *TP53* genes^27^. HCT116 cells express wild type TP53, while DLD1, Caco2, HT29, and SW620 cells express mutant TP53. However, the status of TP53 did not affect MHY-induced cytotoxicity (Fig. S1a, b). Moreover, Caco2 and SW620 cells express wild type PI3KCA but DLD1, HCT116, and HT29 cells express mutant PI3KCA. MHYs decreased cell viabilities regardless of the status of PI3KCA (Fig. S1a, b). Additional studies are needed to gain a better understanding of the mechanisms responsible for different cytotoxic effects of MHYs in various colon cancer cell lines.

It is noteworthy that the activation of the Src signaling pathway can contribute to apoptosis. When Ras and PI3K are inhibited, v-Src generates proapoptotic signals in fibroblasts^11^. In the presence of MHYs, the Ras/ERK signaling pathway was inhibited and yet the Src activity was enhanced (Figs. 3b, c and 4a). Another study reports that c-Src regulates estrogen-induced stress and inhibition of c-Src blocks estrogen-induced apoptosis in breast cancer cells^12^. Moreover, Src can activate the JNK signaling pathway which plays a key role in the intrinsic mitochondrial apoptosis^13,46^. As shown in Fig. 4b, MHYs increased the phosphorylations of JNK and c-Jun suggesting the involvement of the JNK signaling pathway in MHY-triggered apoptosis. Although MHYs activated the Src/JNK pathway, gefitinib did not phosphorylate Src (Fig. 5b). Additionally, PD168393 induced Src phosphorylation (Fig. 5c). Overall, it seems that reversible inhibition of EGFR by gefitinib does not utilize the Src signaling pathway to exert pro-apoptotic effect, while irreversible inhibition of EGFR by PD168393 and MHYs activates Src/JNK signaling pathways to further exert their cytotoxicity. Since MHYs induced neither Src phosphorylation nor apoptosis in EGFR null colon cancer SW620 cells and inhibition of Src activity by a chemical inhibitor or siRNA approach completely blocked MHY-mediated cell death, the cytotoxic effects of MHYs require not only EGFR inhibition but also Src activation.

It will be necessary to perform a further analysis to clarify the mechanism of which MHYs induce Src phosphorylation. Previous studies suggested that ROS or ER stress can trigger apoptosis and activate the Src/JNK pathway^38,43,45,47^. Moreover, oxidation of c-Src at Cys245 and Cys487 results in Src activation through the dephosphorylation at the Tyr527 in the C-terminal regulatory region and the phosphorylation at the Tyr416 in the kinase domain, suggesting that oxidative condition is capable of inducing c-Src activation^48^. Consequently, c-Src kinase phosphorylates Cas to generate the Cas–Crk complex, which subsequently activates JNK-mediated apoptosis pathway^49^. Therefore, ROS production is capable of inducing Src activation, followed by JNK activation and subsequent cell death. MHYs induced ROS generation and ER stress, activated the Src/JNK pathway for mitochondrial apoptosis, and ROS scavenger suppressed Src-mediated apoptosis (Fig. 6f).

The underlying mechanism by which MHYs exert anti-cancer activity is described in Fig. 7. EGFR inhibitors can suppress cell proliferation signaling mediated by the Ras/Raf/MEK/ERK, and also elevate the oxidative stress and ROS in cancer cells^41^. When the oxidative stress level surpasses the reduction capacity of cancer cells, cells can undergo apoptosis or necrosis. Increased ROS production also triggers ER stress and mediates the Src/JNK activation which is involved in apoptosis^42,49^. Therefore, MHYs inhibit EGFR phosphorylation and subsequently increase the oxidative stress. Src recognizes oxidative stress and activates the JNK signaling pathway which can trigger apoptosis as evidenced by mitochondrial membrane dysfunction, Cyt *c* release and PARP/caspase-3/-9 activation.

In summary, novel *β*-phenylacrylic acid derivatives have been shown to target KRAS wild-type colon cancer by inhibiting EGFR as well as generating stress responses. The Src/JNK signaling pathway is involved in this mitochondrial apoptotic process suggesting a new role of Src in colon cancer.

## Materials and methods

### Materials

β-Phenylacrylic acid derivatives, MHYs, were synthesized by Dr. Hyung Ryong Moon and the structures of MHY compounds are shown in Fig. 1a. MHY791 is (*Z*)-5-(4-hydroxy-3,5-dimethoxybenzylidene)-2-thioxoimidazolidin-4-one^50^. MHY1036 is (*Z*)-2-acetamido-3-(4-hydroxy-3-methoxyphenyl) acrylic acid. All MHY compounds were dissolved in dimethyl sulfoxide (DMSO, Duchefa biochemie B.V., Haarlem, Netherlands) at 10 mM concentration and were stored at −80 °C until use. The maximum concentration of DMSO did not exceed 0.1% (v/v) after dilution in DMEM or RPMI-1640 (both from Hyclone Inc., Logan, UT, USA) to the working concentration of 10 μM. PD168393 (Cat. # PZ0285) was purchased from Sigma-Aldrich (St. Louis, MO, USA) and gefitinib (Cat. #13166) was purchased from Cayman Chemical (Ann Arbor, MI, USA).

### Cell culture system

The human colon cancer cell lines, Caco2, HT29, and DLD1 were purchased from ATCC (Manassas, VA, USA), and HCT116 was from KCLB (Seoul, Republic of Korea), and the normal colon epithelial cell line NCM460 were obtained from INCELL Corporation (San Antonio, TX, USA). HCT116 and DLD1 cell lines were cultured in RPMI-1640, and Caco2, HT29, and NCM460 were cultured in DMEM. Each medium contained 10% heat inactivated fetal bovine serum (FBS), 100 units/ml penicillin, and 100 μg/ml streptomycin (all from Hyclone Inc.). All cell lines were incubated at 37 °C in a humidified environment with 5% CO_2_. PP2 (Cayman Chemical), Src Tyr kinase inhibitor, was dissolved in DMSO. Stock concentration was 100 μM and stored at −20 °C until use. NAC (Enzo Life Sciences Incs., Farmingdale, NY, USA) was dissolved in 500 mM NaHCO_3_ solution as 500 mM and stored at −20 °C and used as 5 mM. PP2 and NAC were pre-treated in each cell line for 30 min, then MHYs or vehicle (DMSO) was treated for 24 h.

### Caco2 cell permeability assays

Caco2 (9 × 10^3^ cells/well) were seeded on semipermeable surfaces of insert plates (SPL, #37024) and were grown in Dulbecco’s Modified Eagle’s medium with no phenol red (DMEM-NPR, Hyclone, SH30284) containing 10% FBS and 1% P/S for 3 days. MHY 791 or MHY 1036 (100 μM) dissolved in DMEM-NRP was placed in the apical side of cell monolayer and left for 24 h. The concentrations of MHYs in the apical and the basolateral sides were determined by using UV–Vis spectrophotometer at 461 nm (for MHY 791) and 372 nm (for MHY 1036). Accumulation percentage of MHYs in each side of the monolayer is calculated as follows; [*A* or *B*/(*A* + *B*)] x 100. *A*: the amount of MHYs in the apical side, *B*: the amount of MHYs in the basolateral side.

### Docking simulation

Docking simulation was performed using AutoDock Vina 1.1.2 software (The Scripps Research Institute, La Jolla, CA, USA). And receptor PDB files which were used to simulate docking (4WRG, 4WKQ, 1MOX) were downloaded from Protein Data Bank. Ligands (MHY791, MHY1036) were drawn using Chemdraw 12.0 (Cambridge Soft Corporation, Cambridge, MA, USA) and prepared by MGL Tools 1.5.4. Receptor coordinates were calculated using UCSF Chimera 1.10.1. After simulation, the analysis and visual investigation of ligand–protein interactions of docking poses were performed using PyMol v1.7 (Schrodinger LLC, New York, NY, USA). First, 4WRG (free tyrosine kinase domain) was employed to search for binding domains of MHY791, MHY1036. Grid box (docking space) was set to cover whole receptor protein. After docking simulation, the clusters gathered were examined. Also 1MOX (extracellular domain) was employed for simulation using the same method as for 4WRG. Second, 4WKQ (Gefitinib bound tyrosine kinase domain) was employed to analyze alternative binding conformations. Before simulation, native ligand (Gefitinib) was removed from the tyrosine kinase domain and grid box was confined to previous ligand size (size of *x*, *y*, *z* = 20). After simulation, polar hydrogens were added and hydrogen bonds were calculated by using PyMol v1.7. To calculate the binding affinity of Gefitinib, simulation was performed and the docking poses were compared with cocrystal conformation. The conformation most identical to that of cocrystal structure was selected for affinity calculation.

### Immunoblot analysis

Cells were washed with cold phosphate-buffered saline (PBS) (pH 7.5) (Hyclone Inc.), harvested, and incubated in protein extraction solution (ELPIS-biotech, Daejeon, Republic of Korea) containing protease inhibitor cocktail 2 (Sigma-Aldrich) and phosphatase inhibitor cocktails (Roche Diagnostic, Mannheim, Germany). The lysis buffer does not contain Triton X-100 to avoid a disruption of mitochondrial membrane. Cell lysis was facilitated by vortexing and incubation on ice for 1 h. Cell lysates were clarified by centrifugation at 4 °C and 15,000 × *g* for 20 min. Protein (50 μg) from each lysate was loaded onto a 10% sodium dodecyl sulfate–polyacrylamide gel and electrophoresis was performed. Proteins were transferred to polyvinylidene difluoride membranes by using a 500 mA current for 4 h. The membrane was blocked with 5% skim milk or bovine serum albumin in 1× Tris-buffered saline with Tween-20 buffer (TBS-T) for 1 h at RT and then incubated overnight at 4 °C with one of following primary antibodies: β-actin (A5441, Sigma-Aldrich), ATF-6α (sc22799, Santa Cruz), Bcl-xL (#2764 Cell Signaling, Danvers, MA), Bid (#2002, Cell Signaling), Bik (#4592, Cell Signaling), Bim (#2933, Cell Signaling), cleaved caspase-3 (#9661, Cell Signaling), full length caspase-3 (#9665, Cell Signaling), cleaved caspase-9 (#9505, Cell Signaling), full length caspase-9 (#9502, Cell Signaling), Cyt *c* (sc-7159, Santa Cruz, Paso Robles, CA, USA), EGFR (sc-03, Santa Cruz), phosphor-IRE1α (PA1-16927, Thermo Fisher Scientific, Seoul, Republic of Korea), cleaved PARP (#5625, Cell Signaling), PARP (sc-7150, Santa Cruz), phospho-PERK (Abcam, Suite B2304 Cambridge, MA, USA), TFIIB (Bioworld Technology, St. Louis Park, MN, USA), α-tubulin (#2144, Cell Signaling), and phospho-Tyr (601100, BD Biosciences, Ann Arbor, MI, USA). After the membrane was washed for 50 min with 1× TBS-T, membranes were incubated with an appropriate horseradish peroxidase-conjugated secondary antibody (Enzo Life Sciences, NY) for 2 h at RT. Bound antibodies were detected by the enhanced chemiluminescence detection system (Advansta WesternBright^TM^ ECL, Menlo Park, CA).

### Immunofluorescence staining

HT29 cells were seeded in the Nunc™ 154526 Lab-Tek^®^ II chamber Slide™ System (Thermo Fisher Scientific) at a density of 1 × 10^5^ cells/well. After 24 h, cells were treated with vehicle (DMSO), MHY791, or MHY1036 for 24 h. Cells were washed with warm PBS twice and fixed with 10% neutral buffered formalin solution (Sigma-Aldrich) for 30 min. Fixed cells were washed twice with PBS, placed in quenching solution (0.1 M glycine in PBS) for 15 min, and washed with PBS twice. Cells were incubated in blocking and permeabilization solution (1% BSA in PBS and 0.1% Triton X-100) for 1 h at RT. Permeabilized cells were incubated overnight in 1:5000 diluted Cyt *c* antibody at 4 °C. After washing, cells were incubated in a 1:5000 dilution of fluorescein isothiocyanate (FITC) conjugated rabbit IgG-heavy and light chain antibody (Bethyl Laboratories, Montgomery, TX, USA). Finally, cells were mounted using VECTASHIELD^®^ mounting medium with 4′,6-diamidino-2-phenylindole (DAPI, Vector Laboratories, Burlingame, CA, USA). Stained samples were observed by FV10i (Olympus Inc, Center Valley, PA, USA) with image analysis by FV10i fluoview software.

### Immunoprecipitation

Caco2 and HT29 cells were seeded in 100-mm cell culture plates at a density of 5 × 10^6^ cells/plate and incubated for 24 h. Cells were washed with pre-warmed PBS and pre-treated with MHYs in the absence of FBS (serum-free medium, SFM) at 37 °C for 15 min. Ten percent FBS (v/v) or 100 ng/ml EGF was added to cells and incubated for 5 min. Cells were washed twice with cold PBS, harvested by suspension in protein extraction solution, and lysed by vortexing and incubation on ice for 1 h. Five-hundred micrograms of protein was incubated with 10 μl (2 μg) of EGFR antibody (sc-03, Santa Cruz) for 1 h at 4 °C. Twenty microliters of Protein G PLUS-Agarose beads (sc-2002, Santa Cruz) on a rocker platform for overnight at 4 °C. Beads were washed four times with PBS containing phosphatase inhibitor cocktail 2 and proteinase inhibitor cocktail. After the final wash, beads were re-suspended in 1× protein sample buffer (ELPIS-BIOTECH) and fractionated by SDS–PAGE, followed by immunoblotting as described above. Phosphorylation of EGFR was detected by phospho-Tyr (601100, BD Biosciences).

### Measurement of mitochondrial membrane dysfunction

Mitochondrial membrane dysfunction was measured using the Mito-ID^®^ Membrane potential detection kit (ENZ-51018, Enzo Life Sciences Inc.). HT29 cells were seeded at a density of 5 × 10^5^ cells/well in six-well plates. Cells were treated with vehicle (DMSO), MHY791, or MHY1036 by dilution in culture medium. After 24 h, all samples were prepared according to the manufacturer’s instructions. For detection of mitochondrial membrane dysfunction, samples were stained with Mito-ID MP detection reagent for 15 min. Samples were monitored using FACS (Accuri C6, BD Biosciences). The term “de-energized mitochondria” was used to express reduction of mitochondrial membrane potential due to mitochondrial dysfunction during apoptosis. If mitochondria membrane potential is decreased by mitochondria dysfunction, JC-1 exists as monomer in cytosol expressing green fluorescence. The intensity of green fluorescence was measured to compare JC-1 levels between control and MHY groups.

### Mouse xenograft model of human colon cancer

The animal study protocols used in this study were approved by the Institution Animal Care and Use Committee of Pusan National University (PNU-IACUC, Busan, Republic of Korea) for ethical procedures and scientific care (Approval number PNU-2015-1043). Female BALB/c immunodeficient mice (4 weeks of age) were purchased from Orientbio (Sungnam, Gyounggi, Republic of Korea). Mice were acclimated to the animal care facility for 1 week. Mice were housed in an air-conditioned atmosphere under a 12-h light and dark cycle and had free access to water and rodent chow (Orientbio). Mice were inoculated in the flank with 1 × 10^6^ HT29 cells by injection. Seven days after inoculation, MHY791 or MHY1036 (3 mg/kg in 100 μl) or DMSO was administered at the tumor sites. Tumor size and body weight were measured each day with calipers and tumor volume was calculated as (length × width^2^) × 0.5^51^. Seven days after the first trial with MHYs or vehicle, mice were euthanized. Tumors were excised and tumor size and weight were measured.

### MTT assay

Cell viability was assessed by different methods including the 3-(4,5-dimethylthiazol-2-yl)-2,5-diphenyltetrazolium bromide (MTT, Sigma-Aldrich) assay. Cells were seeded in 96-well culture plates at a density of 5 × 10^3^ cells/well. All assays were performed in quintuplicate. Each cell line was cultured in the media as mentioned above. At 24 h after seeding, each MHY compound was added at the indicated concentrations for 24 h. After removing the growth medium, the cells were incubated with 0.5 mg/ml of MTT at 37 °C for 4 h. Live cells formed insoluble formazan that was dissolved in DMSO. Absorbance was measured at 540 nm by an iMark™ Microplate Absorbance Reader (Bio-Rad Laboratories, Hercules, CA, USA).

### ROS detection analysis

ROS development during 30 min after MHY compounds treatment was measured by total ROS detection kit (ENZ-51011, Enzo life sciences Inc.). HT29 cells were seeded in six-well plates at a density of 1 × 10^6^ cells/well. After 24 h, the positive control was treated with the ROS inducer, pyocyanin, and the negative control with ROS inhibitor, NAC. Treated groups were treated with vehicle (DMSO), MHY791 or MHY1036. All procedures were conducted according to the manufacturer’s instructions. After treatment, cells were trypsinized and resuspended in an ROS detection solution at 37 °C in dark for 30 min. Results were monitored by FACS. ROS generation at other time point was measured by dichlorofluorescein assay. HT29 cells were seeded in 96-well clear bottom black microplates (Corning, New York, NY, USA) at a density of 5000 cells/well. Twenty four hours after seeding, vehicle (DMSO), MHY791, and MHY1036 were treated. Pyocyanin was supplied as lyophilized powder in the kit and reconstituted in 100 µl *N,N*-dimethylformamide (DMF, TCI America, OR, USA) to yield a 10 mM. The final concentration of pyocyanin was 200 µM. 2′,7′-dichlorodihydrofluorescein diacetate (Molecular Probes, Eugene, OR, USA) was diluted in PBS, and treated as working concentration of 0.125 mM. Fluorescence was monitored by microplate reader (Berthord, Bad wildbad, Germany). Fluorescence microplate reader was set as excitation filter at 485 nm and as emission filter at 535 nm. The data were recorded 5 min apart during 30 min.

### Ras activation assay

Ras activation assay was performed with Ras activation kit (ADI-EKS-460, Enzo life sciences Inc.). Caco2, HT29, and HCT116 cells were seeded at density of the 4 × 10^6^ cells/plate in 100 mm plate. Each plate was treated with vehicle (DMSO) or MHY791 or MHY1036 at the final concentration of 10 μM. After 24 h, protein lysates were prepared as mentioned in the manual. 500 μg of protein lysates were added into immobilized glutathione resin which bind to Glutathione S-transferase-Raf1-Ras-binding domain, and incubated the reaction mixture at 4 °C for 1 h on the rocker. After that, beads were washed, boiled with 2× SDS sample buffer at 95 °C for 5 min. Finally, all samples were analyzed by immunoblot analysis as mentioned above.

### siRNA transfection

Src small interfering RNA (siRNA, 5′-GAGAACCUGGUGUGCAAAG-3′) and scrambled siRNA (SN-1002) were purchased from Bioneer (Daejeon, Republic of Korea). Lipofectamine 3000 transfection reagent (L3000015), and opti-MEM (31985070) were purchased from Sigma-Aldrich. Cells were seeded at a density of 1 × 10^6^ cells/well in six-well plates to be 70–80% confluent. After 24 h, lipofectamine 3000 reagent was diluted in opti-mem and mixed at 4 °C, and 5 μg of siRNAs are diluted and mixed in opti-mem. Then, diluted siRNAs and diluted lipofectamine 3000 were mixed at 1:1 ratio and incubated for 5 min at RT. Finally, siRNA and lipid complex were added to cell, and 4 h after transformation, 10% (v/v) of FBS was added. Transfected cells were incubated at 37 °C for 20 h.

### TUNEL assay

On the final day of mouse xenograft experiment, excised tumors from sacrificed mice were fixed in 10% neutral buffered formalin solution. Tumors were embedded in paraffin and sectioned in 12 μm. TUNEL assay for apoptosis detection was undertaken in ABION CRO (Seoul, Republic of Korea). The results were analyzed by microscope (Olympus Corporation, Tokyo, Japan) and Moticam 3.0 MP Color Digital Camera (Motic, Causeway Bay, Hong kong).

### Wash-out assay

Cells were seeded at a density of 1 × 10^6^ cells/well in six-well plates. Cells were pre-treated with MHY791, MHY1036, or PD168393 in SFM for 1 h and then cells were washed twice in warm PBS and SFM to remove MHYs or PD168393. This process was repeated every 2 h until 6 h from the first wash. Cells were then stimulated with EGF (100 ng/ml) for 5 min and harvested. All samples were analyzed by immunoprecipitation as described above.

### Statistical analysis

Statistical analysis was performed using GraphPad Prism version 6.0 for windows (GraphPad Software, San Diego, CA, USA). Data are presented as the mean ± SD. Group analysis was compared by one-way or two-way ANOVA followed by multiple-comparison Bonferroni *t*-test or Tukey post hoc test to assess differences between groups. Otherwise, paired and two-tailed Student’s *t*-tests were used to compare results. A *p*-value of less than 0.05 was considered statistically significant.

## Electronic supplementary material

Supplementary Figure S1 legend

Supplementary Figure S1
