# Supplementary material for: Novel β-phenylacrylic acid derivatives exert anti-cancer activity by inducing Src-mediated apoptosis in wild-type KRAS colon cancer
Source: Cell Death Dis. 2018 Aug 29;9(9):877. doi: 10.1038/s41419-018-0942-x (PMC6115383; doi:10.1038/s41419-018-0942-x)
Supplement: Supplementary file 2 — Supplementary Figure S1 [file 41419_2018_942_MOESM2_ESM.doc]

**Supplementary Fig. 1. Effects of MHY791 and MHY1036 on cell viability in human colon cancer cell lines.** DLD1, HCT116, Caco2, and HT29 cells were incubated with 10 μM (a) MHY791 (n=5) or (b) MHY1036 (n=5) for 24 h and cell viabilities were measured by MTT assays. Data are presented as ratios relative to the vehicle control (set at 100%). Data are shown as mean ± SD. Statistical significance was determined by paired two-sided *t*-test. Statistical significance is indicated as **P <0.01, ****P <0.0001.
